# Selection History Modulates Working Memory Capacity

**DOI:** 10.3389/fpsyg.2016.01564

**Published:** 2016-10-07

**Authors:** Bo-Cheng Kuo

**Affiliations:** Department of Psychology, National Taiwan UniversityTaipei, Taiwan

**Keywords:** attention, limited capacity, selection history, top-down control, working memory

## Abstract

Recent studies have shown that past selection history affects the allocation of attention on target selection. However, it is unclear whether context-driven selection history can modulate the efficacy of attention allocation on working memory (WM) representations. This study tests the influences of selection history on WM capacity. A display of one item (low load) or three/four items (high load) was shown for the participants to hold in WM in a delayed response task. Participants then judged whether a probe item was in the memory display or not. Selection history was defined as the number of items attended across trials in the task context within a block, manipulated by the stimulus set-size in the contexts with fewer possible stimuli (4-item or 5-item context) or more possible stimuli (8-item or 9-item context) from which the memorized content was selected. The capacity measure (i.e., the *K* measure) was estimated to reflect the number of items that can be held in WM. Across four behavioral experiments, the results revealed that the capacity was significantly reduced in the context with more possible stimuli relative to the context with fewer possible stimuli. Moreover, the reduction in capacity was significant for high WM load and not observed when the focus was on only a single item. Together, these findings indicate that context-driven selection history and focused attention influence WM capacity.

## Introduction

Working memory (WM) allows us to hold and manipulate information that is relevant to our current task goals and plan objectives over a short period of time (Baddeley, 1986). However, the capacity of WM is highly limited (Luck and Vogel, 1997; Cowan, 2001) – only about three or four pieces of information can be retained at any given time. The underlying processes and mechanisms that support the constraints of capacity are largely investigated in the WM literature (Jonides et al., 2008; Edin et al., 2009; Luck and Vogel, 2013; Ma et al., 2014; D’Esposito and Postle, 2015; Stokes, 2015; Postle, 2006, 2015). Evidence from this body of research has revealed that attention is of importance in controlling the contents of WM. Whereas past selection history affects the allocation of attention on target selection (Awh et al., 2012; Belopolsky and Awh, 2016), it remains unexplored whether selection history can influence WM. The goal of this study aims to investigate whether the history of attentional processing can modulate WM capacity.

Accumulating evidence has revealed that WM capacity is modulated by attention (Awh and Jonides, 2001; Gazzaley, 2011; Gazzaley and Nobre, 2012). These studies show that top-down attention serves as the selective mechanism for encoding task-relevant information into WM (Schmidt et al., 2002; Murray et al., 2011) and introducing bias toward cued items during WM maintenance (Griffin and Nobre, 2003; Lepsien and Nobre, 2007; Kuo et al., 2011, 2012, 2014; Astle et al., 2012; Pertzov et al., 2013; Rerko and Oberauer, 2013). These findings suggest that attention mechanisms may determine the total amount of information that can be effectively stored in WM (Edin et al., 2009; Oberauer and Hein, 2012). For example, evidence from electrophysiological recordings in humans revealed that these top-down mechanisms are biased toward a subset of relevant items and prevented irrelevant distractors from entering WM (Vogel et al., 2005).

Recently, an inspiring attention framework suggests that the history of attentional control affects the selective biasing of information processing (Awh et al., 2012; Belopolsky and Awh, 2016). In this framework, recent history of attentional selection (i.e., selection history) elicits *a lingering consequence of past selection episodes or goals* (Awh et al., 2012, p. 437). The lingering effect of past selection can form a bias toward recently activated representations or templates, exerting a powerful influence on the selection priority for a target item to accomplish the current task. Behavioral and electrophysiological studies in humans have shown that selection history may cause an inter-trial priming effect for the processing of the predefined target features (Maljkovic and Nakayama, 1994; Hillstrom, 2000; Fecteau and Munoz, 2003; Wolfe et al., 2003; Geyer et al., 2006, 2011; Eimer et al., 2010; Kristjánsson and Campana, 2010; Brascamp et al., 2011; Töllner et al., 2012; Gokce et al., 2014; Feldmann-Wüstefeld and Schubö, 2016). For example, when participants were required to judge the shape of a pop-out color target amid multiple distractors, they showed faster search times when the target color repeated the one shown in the preceding trial relative to the non-repeated trials (Maljkovic and Nakayama, 1994). A recent study using a flanker task also showed that the context-driven selection history modulated distractor processing so that the processing mode in one block persisted in the subsequent block (Yeh et al., 2014). These findings indicated that an implicit setting of selection, inter-trial repetition or consistent processing mode across trials, can be embedded within a task context to influence the perceptual processing of a target item.

Given the influences of selection history on attentional control and the importance of attention in WM, selection history should influence WM performance. Yet, no prior study has investigated the impact of selection history on WM. This study aims to test the influences of context-driven selection history on WM capacity. Participants performed a delayed response WM task for colors (Experiments 1 and 3) and shapes (Experiments 2A and 2B) with both high (three items in Experiments 1 and 2; four items in Experiment 3) and low WM loads (one item). They were first presented with one item (low WM load) or three/four items (high WM load) within a memory array, and following a short retention interval, they responded to a test probe based on the retained information. Selection history was operationally defined as the number of items that had been attended across trials in a block, manipulated by the stimulus set-size (4-item and 8-item contexts in Experiments 1 and 2, and 5-item and 9-item contexts in Experiment 3) from which the memorized content was selected. Although the memory array always contained one or three/four items, the WM task was performed in two different contexts. In one context (4-item or 5-item), fewer memorized contents from the previous trials could interfere with the processing of task-relevant items in the current trial. In another context (8-item or 9-item), more representations from previous trials could interfere with stimulus processing in the current trial. The lingering effect refers to the influence of previous memorized content on the efficacy of attentional control on performing the WM task. Attentional control should be less effective when more representations have been activated unless only one item needs to be encoded in the WM task. Thereby, I hypothesized that WM capacity would be reduced in the context with more possible stimuli unless attention focuses on a single item to protect it from interference caused by the lingering effect of previous trials.

## Materials and Methods

### Participants

In total, 66 healthy volunteers participated in this study. Twenty volunteers participated in Experiment 1 (11 females, age range 20–26 years, mean age = 22.10). Sixteen different volunteers participated in Experiment 2A (9 females, age range 20–27 years, mean age = 22.06) and 14 different volunteers participated in Experiment 2B (8 females, age range 20–26 years, mean age = 20.86). Finally, another group of sixteen volunteers participated in Experiment 3 (10 females, age range 20–23 years, mean age = 21.06). All participants were right-handed, according to the Edinburgh handedness inventory (Oldfield, 1971); they had normal or corrected-to-normal visual acuity, provided informed written consent prior to the study and were financially reimbursed for their time. All experimental methods and procedures received ethical approval from the Research Ethics Office of National Taiwan University.

## Experiment 1

### Method

#### Stimuli

Stimuli were presented with Presentation software (Neurobehavioral Systems, Albany, NY, USA). Eight color stimuli were selected for this experiment (blue, yellow, green, red, pink, cyan, brown, and gray). The luminance value was measured for each color (blue: 36.8 cd/m^2^; yellow: 143.0 cd/m^2^; green: 63.6 cd/m^2^; red: 38.5 cd/m^2^; pink: 85.9 cd/m^2^; cyan: 93.2 cd/m^2^; brown: 8.1 cd/m^2^; gray: 21.5 cd/m^2^). Each color square stimulus subtended a visual angle of approximately 1.3° × 1.3° (edge-to-edge) and was positioned randomly in one of four possible peripheral locations of an invisible 2 × 2 matrix that subtended approximately 3.74° (vertical) × 4.64° (horizontal). A black (luminance value: 0.05 cd/m^2^) background was used throughout the experiment.

#### Design and Procedure

**FIGURE 1:**
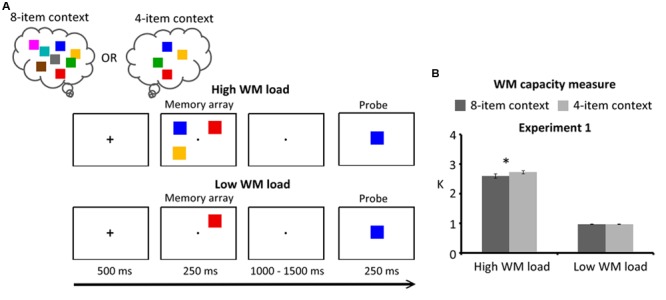
**(A)** Schematic illustration of the delayed response task of Experiment 1. Participants viewed a memory array consisting of three colors (high WM load) or one color (low WM load) at the beginning of the trial (250 ms duration). Following a randomized retention interval (1000–1500 ms duration), a probe item was centrally presented for 250 ms. Participants were instructed to indicate whether the remembered item was present (50%) or absent (50%). Selection history was defined as the number of items that have been activated in the task context (e.g., 4-item and 8-item). These two task contexts were presented in a block design. The order of task contexts was counterbalanced across participants. Half of the participants started with the 8-item context, and the other half started with the 4-item context. **(B)** WM capacity (*K* estimates) for Experiment 1. Error bars represent standard errors of the means. WM: working memory.

The experimental design followed a 2 (task context: 8-item and 4-item) × 2 (WM load: high load and low load) × 2 (response type: target present and target absent) within-subjects factorial design. In the 8-item context, all eight colors were used in a block and the memorized content (three items or one item) on each trial was randomly selected from this set. In the 4-item context, a set of four colors was used in a block and the memorized content on each trial was randomly selected from this set. Participants were instructed to remember the memorized content and match a test probe with the to-be-remembered item(s) after a retention interval. The order of task contexts was counterbalanced across participants: half of the participants started with four blocks of the 8-item context, and the other half started with four blocks of the 4-item context. Participants were not informed about the manipulation of the task contexts. The task procedure is shown in **Figure 1A**.

Each trial began with a centrally displayed fixation cross (500 ms duration). After the cross, the participants viewed a memory array consisting of one or three peripheral colors for 250 ms. Participants were instructed to remember all the colors within the memory array. Following a randomized retention interval (1000–1500 ms duration), a probe item was centrally presented for 250 ms. Participants were instructed to indicate, using their right hand, whether this item had been present in the preceding memory array or not. On half of the trials, the probe stimulus matched the memorized content. They were instructed to respond as accurately and quickly as possible and maintain fixation on a small fixation marker at the center of the screen during the experimental trials. The interval between trials, which included a 1000-ms response period, varied between 2000 and 3000 ms.

The participants were seated approximately 57 cm from a CRT monitor. Prior to the formal experiment, the participants were given both written and verbal instructions about the task requirements. They first completed one practice block (16 trials) to ensure that they could perform the task as instructed. The formal experiment consisted of eight blocks of 48 trials, which participants could self-initiate (four blocks for each task context). For each block and task context, WM load and response types were intermixed in a randomized and unpredictable order. There were 384 trials in total (48 target-present and 48 target-absent trials in each task context and WM load condition). The total experimental time for each participant was approximately 45 min.

#### Behavioral Analysis

WM capacity (Pashler–Cowan *K* measure) (Pashler, 1988; Cowan, 2001) was analyzed by a repeated-measures analysis of variance (ANOVA) with the following two factors: task context (8-item and 4-item) and WM load (high load and low load). The *K* measure was calculated using the following equation: *K* = *S* (set size of the memory array) × (hit rate - false alarm rate). For the *K* measure, the hit rate was defined as the conditional probability that the participants responded “target-present” when the target was presented and the false-alarm rate was defined as the conditional probability that the participants responded “target-present” when the target was absent. Moreover, the hit rates and false alarm rates were each analyzed by a 2 (task context: 8-item and 4-item) × 2 (WM load: high load and low load) repeated-measures ANOVA (see Supplementary Material for the results of accuracy and response time).

### Results

The results are summarized in **Figure 1B**. The task context had a significant effect [*F*(1,19) = 4.88, *p* = 0.04], with the 4-item context (1.88 ± 0.20 *K*) having higher *K* measures than the 8-item context (1.77 ± 0.19 *K*). WM load also had a significant effect [*F*(1,19) = 1251.72, *p* < 0.001], with low WM load trials (0.96 ± 0.05 *K*) having lower *K* measures than the high WM load trials (2.66 ± 0.24 *K*). More importantly, a significant interaction between the task context and WM load was found [*F*(1,19) = 4.68, *p* = 0.04]. This interaction showed a reduced WM capacity in the 8-item context (2.59 ± 0.34 *K*) relative to the 4-item context (2.72 ± 0.20 *K*) when three target items were maintained in WM [*F*(1,38) = 9.55, *p* = 0.004]. By contrast, that was not a case for the low WM load trials [*F*(1,38) = 0.03, *p* > 0.25]. The simple main effect of WM load was significantly larger in the 4-item context (1.76 ± 0.18 *K*) than in the 8-item context (1.64 ± 0.30 *K*) [*t*(19) = 2.16, *p* = 0.043].

Analyses of hit rates and false alarm rates showed a significant main effect of WM load on hit rates [*F*(1,19) = 19.56, *p* < 0.001] and on false alarm rates [*F*(1,19) = 14.98, *p* = 0.001]. Hit rates were higher for the low WM load (97.71 ± 2.70%) than the high WM load (93.80 ± 4.73%); higher false alarm rates for the high WM load (5.26 ± 4.65%) than the low WM load (1.82 ± 2.68%). The interaction between task context and WM load was also significant on false alarm rates [*F*(1,19) = 5.53, *p* = 0.028]. This interaction indicated higher false alarm rates in the 8-item context (6.67 ± 6.81%) than the 4-item context (3.85 ± 3.46%) when WM load was high [*F*(1,38) = 8.68, *p* = 0.006]. The results are summarized in **Table 1**.

**Table 1:** Hit rates and false alarm rates (in %) for each condition in Experiments 1–3 (standard deviation for each condition was shown in bracket).

	8-item context		4-item context	
	High WM load	Low WM load	High WM load	Low WM load
**Experiment 1**				
Hit	93.02 (6.77)	97.40 (2.94)	94.58 (4.30)	98.02 (3.06)
False alarm	6.67 (6.81)	1.87 (3.02)	3.85 (3.47)	1.77 (2.89)
**Experiment 2A**				
Hit	65.76 (10.46)	95.44 (7.27)	73.96 (12.34)	96.22 (5.44)
False alarm	29.69 (11.27)	5.47 (9.41)	25.39 (11.91)	3.78 (3.82)
**Experiment 2B**				
Hit	64.43 (12.90)	96.43 (3.96)	73.96 (17.35)	95.24 (3.69)
False alarm	19.20 (9.32)	3.57 (2.99)	13.99 (8.19)	4.02 (3.96)

	**9-item context**		**5-item context**	
	**High WM load**	**Low WM load**	**High WM load**	**Low WM load**

**Experiment 3**				
Hit	86.13 (7.54)	93.75 (6.30)	86.33 (9.57)	97.56 (4.07)
False alarm	24.12 (12.45)	6.35 (4.62)	7.52 (9.77)	2.73 (2.94)

In sum, these data revealed that WM performance was influenced by the task context, resulting in a significant reduction of the WM capacity and an increase of false alarm rates in the 8-item context than in the 4-item context when WM load was high.

## Experiment 2

Previous research has shown that WM capacity may vary across stimulus categories (Alvarez and Cavanagh, 2004). The goal of Experiments 2A and 2B was to replicate the results of Experiment 1 based on a different stimulus category – shape.

### Method

**FIGURE 2:**
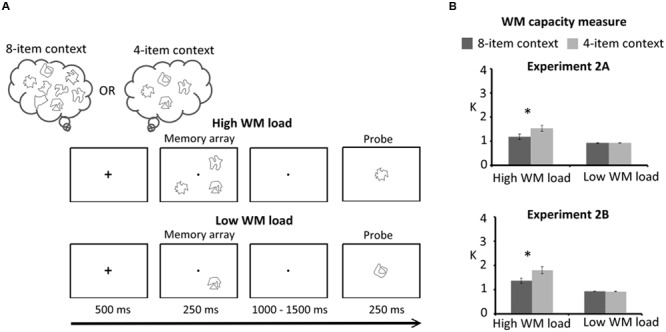
**(A)** Schematic illustration of the delayed response task of Experiments 2A and 2B. Participants were instructed to remember three shapes (high WM load) or one shape (low WM load) within the memory array for a delayed response. The order of task contexts was counterbalanced across participants in Experiment 2A (half of the participants performed the 8-item context first) and Experiment 2B (controlled in a Latin Square design). **(B)** WM capacity (*K* estimates) for Experiment 2A (upper panel) and Experiment 2B (lower panel). Error bars represent standard errors of the means. WM, working memory.

The task of Experiment 2 is illustrated in **Figure 2A**. The methods, including task design, experimental procedure, and behavioral analysis, were identical to those of Experiment 1 except that shapes were used in Experiment 2A. I also tested whether the influences of selection history on WM capacity can be obtained when the order of the task blocks is controlled for each participant in a Latin Square design (e.g., ABBABAAB and BAABABBA, A: 4-item, B: 8-item, counterbalanced across participants) in Experiment 2B.

#### Stimuli

Eight stimuli were selected from a set of one-hundred novel, meaningless closed shape contours developed by Endo et al. (2003). Each stimulus shape subtended a visual angle of approximately 1.3° × 1.3° (edge-to-edge) and was positioned randomly in one of four possible peripheral locations of an invisible 2 × 2 matrix that subtended approximately 3.74° (vertical) × 4.64° (horizontal). All stimulus contours were white. A black background was used throughout the experiment.

## Experiment 2A

### Results

The results are summarized in **Figure 2B** (upper panel). The task context had a significant effect [*F*(1,15) = 13.10, *p* = 0.003], with higher *K* measures in the 4-item context (1.19 ± 0.26 *K*) than the 8-item context (0.99 ± 0.28 *K*). WM load also had a significant effect [*F*(1,15) = 13.66, *p* = 0.002], showing lower *K* measures for low WM load (0.91 ± 0.11 *K*) than for high WM load (1.27 ± 0.43 *K*). Finally, there was a significant interaction between the task context and WM load [*F*(1,15) = 7.77, *p* = 0.01]. This was due to a significant reduction of the WM capacity associated with the 8-item context (1.08 ± 0.48 *K*) relative to the 4-item context (1.46 ± 0.49 *K*) for the high WM load [*F*(1,30) = 20.10, *p* < 0.001] but not for the low WM load [*F*(1,30) = 0.09, *p* > 0.25]. The simple main effect of WM load was significantly larger in the 4-item context (0.53 ± 0.47 *K*) than in the 8-item context (0.18 ± 0.45 *K*) [*t*(15) = 2.79, *p* = 0.014].

The analysis of hit rates showed a significant main effect of task context [*F*(1,15) = 7.92, *p* = 0.013] and a significant main effect of WM load [*F*(1,15) = 136.81, *p* < 0.001], showing higher hit rates in the 4-item context (85.09 ± 8.02%) than the 8-item context (80.60 ± 7.09%) and higher hit rates for the low WM load (95.83 ± 6.12%) than the high WM load (69.86 ± 9.81%). The interaction between task context and WM load on hit rates was also significant [*F*(1,15) = 6.17, *p* = 0.024]. This interaction indicated higher hit rates in the 4-item context (73.96 ± 12.34%) than the 8-item context (65.76 ± 10.46%) when the WM load was high [*F*(1,30) = 14.08, *p* = 0.001]. On false alarm rates, there was a significant main effect of WM load [*F*(1,15) = 85.54, *p* < 0.001], showing higher false alarm rates for the high WM load (27.54 ± 10.08%) than the low WM load (4.62 ± 5.82%).

In sum, these behavioral results confirmed that task context influenced WM performance. WM capacity and hit rates were reduced in the 8-item task context where eight shapes had been attended across trials within a block. This effect of task context was significant only when WM load was high.

## Experiment 2B

### Results

Similar results to Experiments 1 and 2A were found on WM capacity (**Figure 2B**, lower panel). The task context was found to have a significant effect with higher *K* measures [*F*(1,13) = 13.60, *p* = 0.003] in the 4-item context (1.36 ± 0.30 *K*) than for the 8-item context (1.14 ± 0.21 *K*). WM load was also found to have a significant effect [*F*(1,13) = 33.66, *p* < 0.001] with lower *K* measures on low load trials (0.92 ± 0.06 *K*) than on high load trials (1.58 ± 0.45 *K*). Finally, a significant interaction between the task context and WM load was observed [*F*(1,13) = 16.59, *p* = 0.002], indicating a significant reduction of WM capacity in the 8-item context (1.36 ± 0.40 *K*) relative to the 4-item context (1.80 ± 0.57 *K*) for high WM load [*F*(1,26) = 30.10, *p* < 0.001] but not for low WM load [*F*(1,26) = 0.04, *p* > 0.25]. The simple main effect of WM load was significantly larger for the 4-item context (0.89 ± 0.55 *K*) than for the 8-item context (0.43 ± 0.39 *K*) [*t*(13) = 4.07, *p* = 0.001].

The analysis of hit rates showed a significant main effect of task context [*F*(1,13) = 5.49, *p* = 0.034] and a significant main effect of WM load [*F*(1,13) = 62.26, *p* < 0.001], showing higher hit rates in the 4-item context (84.60 ± 9.53%) than the 8-item context (80.43 ± 7.52%) and higher hit rates for the low WM load (95.83 ± 3.47%) than the high WM load (69.20 ± 13.90%). The interaction between task context and WM load was also significant [*F*(1,13) = 9.48, *p* = 0.009]. This interaction arose because hit rates were higher in the 4-item context (73.96 ± 17.35%) than the 8-item context (64.43 ± 12.91%) when the WM load was high [*F*(1,26) = 14.66, *p* = 0.001] whereas the context effect was not significant when WM load was low. The analysis of false alarm rates showed a significant main effect of task context [*F*(1,13) = 8.14, *p* = 0.013] and a significant main effect of WM load [*F*(1,13) = 29.07, *p* < 0.001], showing higher false alarm rates in the 8-item context (11.38 ± 5.29%) than the 4-item context (9.00 ± 4.12%) and higher false alarm rates for the high WM load (16.59 ± 8.32%) than the low WM load (3.79 ± 3.21%). The interaction between task context and WM load was also significant [*F*(1,13) = 11.28, *p* = 0.005]. This interaction indicated higher false alarm rates in the 8-item context (19.20 ± 9.32%) than the 4-item context (13.99 ± 8.19%) for the high WM load [*F*(1,26) = 19.30, *p* < 0.001].

Together, these results confirmed that task context driven by the history of previously attended stimuli influenced WM performance. The results showed a reduction of WM capacity and hit rates in the 8-item context relative to the 4-item context, with high WM load when the block order for the task context was controlled. In contrast to the results of Experiment 2A, context also influenced false alarm rates with higher rates in the 8-item context for high WM load in Experiment 2B.

## Experiment 3

The results from the previous experiments clearly showed that context-driven selection history can influence WM capacity, especially with high WM load. It is noted that high WM load was three items in these three experiments. The goal of Experiment 3 aimed to test whether the influences of selection history on WM capacity can also be observed when the memory set-size was increased to reach the limit in capacity – four items (Luck and Vogel, 1997; Cowan, 2001) for high WM load.

### Method

**FIGURE 3:**
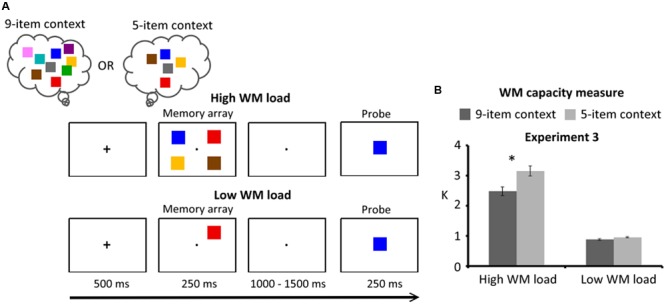
**(A)** Schematic illustration of the delayed response task of Experiment 3. Participants were instructed to remember four colors (high WM load) or one color (low WM load) within the memory array for a delayed response. Selection history was defined as the number of items that have been activated in the task context (e.g., 5-item and 9-item). **(B)** WM capacity (*K* estimates) for Experiment 3. Error bars represent standard errors of the means. WM, working memory.

The task of Experiment 3 is illustrated in **Figure 3A**. The methods, including task design, experimental procedure, and behavioral analysis were identical to those of Experiment 1 with the following exceptions:

#### Stimuli

Nine color stimuli were selected for this experiment (blue, yellow, green, red, pink, cyan, brown, gray, and purple).

#### Design and Procedure

The experimental design followed a 2 (task context: 9-item and 5-item) × 2 (WM load: high load and low load) × 2 (response type: target present and target absent) within-subjects factorial design. Four colors were presented within the memory array for high WM load. In the 9-item context, all nine colors were used in a block and the memorized content (four items and one item) on each trial was randomly selected from this set. In the 5-item context, a set of five colors was used in a block and the memorized content on each trial was randomly selected from this set.

The formal experiment consisted of eight blocks of 64 trials, which participants could self-initiate (four blocks for each task context). For each block and task context, WM load and response types were intermixed in a randomized and unpredictable order. There were 512 trials in total (64 target-present and 64 target-absent trials in each task context and WM load condition). The total experimental time for each participant was approximately 60 min.

### Results

The results were illustrated in **Figure 3B**. The task context had a significant effect with higher *K* measures [*F*(1,15) = 38.36, *p* < 0.001] in the 5-item context (2.05 ± 0.36 *K*) than for the 9-item context (1.68 ± 0.32 *K*). WM load also had a significant effect [*F*(1,15) = 220.84, *p* < 0.001] with lower *K* measures on low load trials (0.91 ± 0.07 *K*) than on high load trials (2.82 ± 0.58 *K*). Finally, a significant interaction between the task context and WM load was found [*F*(1,15) = 27.97, *p* < 0.001], indicating a significant reduction of WM capacity in the 9-item context (2.48 ± 0.57 *K*) relative to the 5-item context (3.15 ± 0.67 *K*) for high WM load [*F*(1,30) = 66.19, *p* < 0.001] but not for low WM load [*F*(1,30) = 0.81, *p* > 0.25]. The simple main effect of WM load was significantly larger for the 5-item context (2.20 ± 0.62 *K*) than for the 9-item context (1.61 ± 0.50 *K*) [*t*(15) = 5.29, *p* < 0.001].

The analysis of hit rates showed a significant main effect of WM load [*F*(1,15) = 61.16, *p* < 0.001], showing higher hit rates for the low WM load (95.65 ± 4.30%) than the high WM load (86.23 ± 7.42%). The analysis of false alarm rates showed a significant main effect of task context [*F*(1,15) = 46.12, *p* < 0.001] and a significant main effect of WM load [*F*(1,15) = 40.54, *p* < 0.001], showing higher false alarm rates in the 9-item context (15.23 ± 8.05%) than the 5-item context (5.13 ± 5.93%) and higher false alarm rates for the high WM load (15.82 ± 9.78%) than the low WM load (4.54 ± 3.40%). The interaction between task context and WM load was also significant [*F*(1,15) = 22.31, *p* < 0.001]. This interaction indicated higher false alarm rates in the 9-item context (24.12 ± 12.45%) than the 5-item context (7.52 ± 9.77%) for the high WM load [*F*(1,30) = 62.82, *p* < 0.001].

In sum, these results confirmed that task context can affect WM performance when the memory set-size increased, indicating a reduction of WM capacity and an increase of false alarm rates in the 9-item context relative to the 5-item context.

## Discussion

Goal-directed behaviors depend upon the allocation of attention toward a subset of relevant information from the external environment and within the internal representations (Awh and Jonides, 2001; Chun et al., 2011; Gazzaley, 2011; Gazzaley and Nobre, 2012). A growing body of evidence has revealed that the context-driven selection history of attentional deployment can generate a lingering bias in selection (Eimer et al., 2010; Kristjánsson and Campana, 2010; Geyer et al., 2011; Awh et al., 2012; Belopolsky and Awh, 2016; Jost and Mayr, 2016). The present study highlighted the influences of selection history on the efficacy of attentional control on WM representations and affect WM capacity in a delayed response task. The context-driven selection history was defined as the number of items that had been attended across trials within a block while performing the WM task. On each trial of different blocks, one (low WM load) or three/four items (high WM load) were randomly selected from a subset of fewer possible stimuli (4-item or 5-item context) or more possible stimuli (8-item or 9-item context) as the to-be-remembered item(s). Participants were instructed to match a test probe with the memorized content after a retention interval and were not informed about the manipulation of the task context. The main finding was that WM capacity was significantly reduced in the context with more possible stimuli relative to the context with fewer possible stimuli. More importantly, this reduction in capacity was observed when WM load was high and was not observed when attention was focused on only one item for WM maintenance. Finally, this detrimental effect in capacity was obtained when the memory set-size reached the capacity limit, e.g., four objects, in WM.

Prior research has examined the mechanisms underlying the impact of selection history on attentional allocation at the perceptual level. These investigations demonstrated that context-driven attention allocation elicited inter-trial priming in visual search tasks (Hillstrom, 2000; Fecteau and Munoz, 2003; Wolfe et al., 2003; Geyer et al., 2006; Becker et al., 2009; Kristjánsson and Campana, 2010; Belopolsky and Awh, 2016). Maljkovic and Nakayama (1994) proposed that the search history of attention-driving features influences search in the subsequent trials. Such inter-trial priming can facilitate the perceptual processing of the search targets and yield selection benefits in response times on repeated trials compared with non-repeated trials. A similar inter-trial priming effect was found in a recent study in which participants’ response to a target singleton was interfered with by an irrelevant color singleton (Theeuwes and Burg, 2011). However, this interference was diminished when the selected feature remained the same from one trial to the next trial. Inter-trial priming could accumulate, through repeating the same spatial layout of stimuli across blocks, to guide spatial attention to the target location and facilitate target search (Chun and Jiang, 1998; Olson and Chun, 2001). This contextual cueing benefit was observed even though explicit memory performance measured by recognition was at the chance level.

Recent electrophysiological studies in humans that exploited an event-related potential marker of attentional selection (e.g., N2pc) (Luck and Hillyard, 1994; Eimer, 1996) in a visual search task provided neural evidence in support of inter-trial priming (Töllner et al., 2012; Gokce et al., 2014). For example, the onset latency of N2pc was delayed when target and distractor colors were changed compared to when they were repeated (Eimer et al., 2010). The subcomponent of the N2pc (e.g., Nd, the negative part of the N2pc contralateral to distractors) was also influenced by the repetition of distractor feature (Feldmann-Wüstefeld and Schubö, 2016). The repetition of distractors can decrease attentional capture by task-irrelevant stimuli in the blocks where participants can expect a specific distractor color compared to the blocks where participants cannot predict the distractor color. Together, these results suggest that the repetition of a feature attribute across trials allows the mechanisms of attention more efficient processing.

The implicit and autonomous influences of selection history on target processing have also been observed in the research line of statistical regularities (Chun and Turk-Browne, 2007; Turk-Browne et al., 2010; Umemoto et al., 2010). In these studies, statistical regularity was usually manipulated in a stream of items with embedded sequences so that certain stimuli precede or follow with other stimuli repeatedly. Selection history in this experimental context refers to the contingency of stimulus arrangement across trials. The extraction of the regularities or patterns can bias the allocation of attention in guiding goal-directed behaviors and thus modulate perceptual and mnemonic operations. For example, regularities triggered implicit perceptual anticipations for perceiving face and scene stimuli (Turk-Browne et al., 2010). In this functional magnetic resonance imaging study, sequential contingencies (e.g., paired images) were embedded in a continuous stream of visual images while participants performed categorical responses. Although participants were not aware of the existence of the trial structure, significant neural activity was observed to the predictive stimuli in the learning-related brain area (e.g., hippocampus).

Regularities also facilitated the encoding of the to-be-remembered items in the location where the targets were presented more likely, even though the participants were not aware of the contingency in a WM task (Umemoto et al., 2010). A recent study also showed that memory of a stream of objects in a structured sequence (e.g., triplet element) based on temporal co-occurrence was better than memory of the objects in random sequences (Otsuka and Saiki, 2016). In this study, the regularities from the structured sequence induced implicit allocation of attention toward the triplets and improved memory performance for each triplet constituent. Moreover, they showed that the benefits of regularities were eliminated when an unexpected distractor was inserted in the structured sequence of the triplets. The benefits of statistical regularities on memory support the notion that the history of stimulus contingency across trials influences memory performance without awareness of the contingency.

The current experimental context differs from those adopted in the study of inter-trial priming and the study of statistical regularity. The memorized content was randomly selected on each trial so that inter-trial priming could not function effectively. No structured sequence was embedded across trials so that statistical regularity could not be formed. Selection history, in a broad sense, refers to past episodic traces of attended representations for accomplishing the WM task goal. A small number of representations were activated in the 4-item/5-item context whereas a large number of representations were activated in the 8-item/9-item context. The history was implicitly accrued in the context for performing the memory task. The effect of context on WM capacity is history-driven, that is not relevant to the current top-down behavioral goal and is not related to stimulus-driven aspects of attentional processing. For the delayed responses, the participants were always presented with a memory array that consisted of one or three/four target items. They were not informed about the manipulation of the task contexts and the task required no explicit knowledge of the past trials. There was no visual information other than the WM targets within the memory array. This experimental design is important for ensuring that the observed effects of selection history are unlikely to be the results of perceptual interferences from the visual cues or distractors. The novel finding of the impact of selection history on WM capacity is significant.

The results of the current study showed null effect when only a single item was focused on and maintained in WM. The attentional demand for encoding one item in WM is relatively low and a single item can be effectively processed and represented in WM. This finding supports the notion that only one item can be focused in attention for selecting goal-directed responses (Oberauer, 2002; Olivers et al., 2011). In this case, attentional mechanisms can effectively modulate this focused representation and resolve the competition from the previously memorized items. Thus, whether the context contained a small or large number of previously memorized items does not matter. Alternatively, this null effect of selection history for the low WM load may result from a ceiling effect as the task is relatively easy.

When WM load was high with three or four items, selection history showed a detrimental effect on WM capacity. In the context with fewer possible stimuli, experience accrued to build episodic traces of four or five different stimuli that had been attended and tagged as task-relevant target templates for WM decision across trials. When a display of three/four items was shown on a trial, only one once-attended target template did not occur. Participants may adopt a discarding strategy to perform high WM load trials by remembering which item was not shown in the memory array. For example, if red, green, blue and yellow were the possible stimuli in the 4-item context, and the to-be-remembered items in a given trial were red, green and blue, participants might just remember “it’s not yellow.” Although participants would need to alter their decision rules between trials for the match and no-match judgments based on WM load, they just have to remember one item in this context. Thus, WM capacity was higher compared with the context that contained more possible items. In the latter context, episodic traces contained eight or nine once-attended target templates across trials. Among these templates, five did not occur in a display of three/four items. The discarding strategy could not function effectively and hence, these five once-attended target templates could cause strong interference on WM operations. This alternative interpretation cannot be fully excluded, because of an increase in false alarm rates for high WM load in the context with more possible items. The discarding strategy, however, cannot explain the results of Experiment 2A because context did not significantly affect false alarm rates. Moreover, this discarding strategy should have led to high *K* measures for the high load condition as only one item (e.g., “it’s not yellow”) is tagged in WM. The results of Experiments 2A and 2B did not support this prediction because *K* was smaller than 2 when three items must be remembered in the 4-item context.

The differential effects of selection history on WM capacity in the current study are in line with the three-embedded components model of WM that distinguishes three states of memory representation in declarative WM (Oberauer, 2002; Lewis-Peacock et al., 2012; Oberauer and Hein, 2012; Larocque et al., 2014). In this model, WM emerges from the interaction between attention and long-term memory (LTM; i.e., activated part of LTM). The region of direct access stores the relevant information selected from the activated LTM representations that is required for immediate access (Woodman et al., 2007; Carlisle et al., 2011). The direct-access region consists of only a subset of elements (e.g., three or four items) and their relationships, which are temporarily bound to the current context. Finally, the state of the focus of attention serves as a selection device. It selects a single item or chunk from the set of information in the direct-access region for action selection. The present results suggest that the once-attended items activate representations in LTM and could cause interference only when multiple items must be retrieved from activated LTM into the region of direct access. The lingering effect does not affect the state in the focus of attention. This theoretical speculation requires future work to examine how the selection history and focus of attention influence WM using various techniques and paradigms. Future research should also test the generalizability of the current results to WM load higher than the capacity limitation of WM (e.g., six items in the memory array).

## Conclusion

The findings from the current study suggest that the lingering effects of selection history for recently attended stimuli can cause strong interferences with currently relevant WM targets and reduce WM capacity, especially when the inter-item competition is strong between recently attended target templates and currently maintained representations. The lingering selection bias is considered an implicit context-driven effect. The present results highlight the influences of the history of selection and focused attention in WM capacity. These findings also bolster the notion that WM representations are highly flexible and susceptible to the different task contexts.

## Author Contributions

The author confirms being the sole contributor of this work and approved it for publication.

## Conflict of Interest Statement

The author declares that the research was conducted in the absence of any commercial or financial relationships that could be construed as a potential conflict of interest.
