# Supplementary material for: Selection History Modulates Working Memory Capacity
Source: Front Psychol. 2016 Oct 7;7:1564. doi: 10.3389/fpsyg.2016.01564 (PMC5053993; doi:10.3389/fpsyg.2016.01564)
Supplement: Supplementary file 1 [file Table_1.pdf]

Supplementary Material

Table 1

Mean accuracy (in %) and mean response time (RT, in ms) for each condition in Experiment 1-3 (standard deviation for each condition was shown in bracket).

	8-item context		4-item context	
	High WM load	Low WM load	High WM load	Low WM load
Experiment 1				
Accuracy (%)				
Target present	93.02 (6.77)	97.40 (2.94)	94.58 (4.30)	98.02 (3.06)
Target absent	93.33 (6.81)	98.13 (3.02)	96.15 (3.47)	98.23 (2.89)
RT (ms)				
Target present	691.40 (131.53)	530.55 (115.76)	661.91 (128.71)	544.82 (157.09)
Target absent	660.05 (98.89)	569.53 (133.76)	625.20 (132.38)	600.99 (139.93)
Experiment 2A				
Accuracy (%)				
Target present	65.76 (10.46)	95.44 (7.27)	73.96 (12.34)	96.22 (5.44)
Target absent	70.31 (11.27)	94.53 (9.41)	74.61 (11.91)	96.22 (3.82)
RT (ms)				
Target present	840.71 (216.99)	656.74 (247.14)	769.47 (219.91)	606.75 (273.79)
Target absent	880.76 (222.75)	690.85 (251.05)	856.10 (222.73)	700.24 (283.34)
Experiment 2B				
Accuracy (%)				
Target present	64.43 (12.90)	96.43 (3.96)	73.96 (17.35)	95.24 (3.69)
Target absent	80.80 (9.32)	96.43 (2.99)	86.01 (8.19)	95.98 (3.96)
RT (ms)				
Target present	866.40 (176.47)	608.50 (188.22)	768.26 (135.70)	573.37 (162.66)
Target absent	867.69 (191.44)	660.72 (183.33)	833.93 (163.95)	640.51 (151.91)
	9-item context		5-item context	
	High WM load	Low WM load	High WM load	Low WM load
Experiment 3				
Accuracy (%)				
Target present	86.13 (7.54)	93.75 (6.30)	86.33 (9.57)	97.56 (4.07)
Target absent	75.88 (12.45)	93.65 (4.62)	92.48 (9.77)	97.27 (2.94)
RT (ms)				
Target present	746.76 (120.38)	641.27 (140.78)	727.56 (194.39)	592.52 (181.58)
Target absent	752.13 (121.75)	677.61 (175.89)	693.01 (171.13)	681.94 (204.75)
